# Automatic multiple-zone rigid-body refinement with a large convergence radius

**DOI:** 10.1107/S0021889809023528

**Published:** 2009-07-16

**Authors:** Pavel V. Afonine, Ralf W. Grosse-Kunstleve, Alexandre Urzhumtsev, Paul D. Adams

**Affiliations:** a Lawrence Berkeley National Laboratory, One Cyclotron Road, BLDG 64R0121, Berkeley, CA 94720, USA; b IGBMC, CNRS-INSERM-UdS, 1 rue Laurent Fries, BP 10142, 67404 Illkirch, France; c Département de Physique, Université Henri Poincaré, Nancy 1, BP 239, Faculté des Sciences et des Technologies, 54506 Vandoeuvre-lès-Nancy, France; d Department of Bioenegineering, University of California Berkeley, CA 94720, USA

**Keywords:** rigid-body refinement, multiple-zone protocols

## Abstract

Rigid-body refinement is the constrained coordinate refinement of one or more groups of atoms that each move (rotate and translate) as a single body. The goal of this work was to establish an automatic procedure for rigid-body refinement which implements a practical compromise between runtime requirements and convergence radius. This has been achieved by analysis of a large number of trial refinements for 12 classes of random rigid-body displacements (that differ in magnitude of introduced errors), using both least-squares and maximum-likelihood target functions. The results of these tests led to a multiple-zone protocol. The final parameterization of this protocol was optimized empirically on the basis of a second large set of test refinements. This multiple-zone protocol is implemented as part of the *phenix.refine* program.

## Abstract 2

Systematic investigation of a large number of trial rigid-body refinements leads to an optimized multiple-zone protocol with a larger convergence radius.

## 1. Introduction

The vast majority of macromolecular crystal structures are solved either with experimental phasing methods (see, for example, Blow & Crick, 1959 ▶; Hendrickson, 1991 ▶) or with the molecular replacement method (Rossmann & Arnold, 2001 ▶, and references therein). In the case of experimental phasing the model is built into an electron density map. The resulting model may contain many local errors, but significant concerted displacements are not expected. In contrast, models obtained *via* molecular replacement or with difference Fourier methods can be systematically displaced. In this situation rigid-body refinement (Booth, 1947*a* ▶,*b* ▶, 1949 ▶; Cochran, 1948 ▶; Scheringer, 1963 ▶; Sussman *et al.*, 1977 ▶; Hoard & Nordman, 1979 ▶; Huber & Schneider, 1985 ▶; Yeates & Rees, 1988 ▶; Derewenda, 1989 ▶; Urzhumtsev *et al.*, 1989 ▶; Driessen *et al.*, 1989 ▶; Yeates & Rini, 1990 ▶; Brünger, 1990 ▶ *a*,*b* ▶, 1991 ▶; Castellano *et al.*, 1992 ▶; Noble *et al.*, 1993 ▶; Navaza, 2001 ▶; Tronrud, 2004 ▶; McCoy, 2007 ▶; Lebedev *et al.*, 2008 ▶) is a powerful method for correcting potentially large systematic displacements. Outside the field of crystallography, rigid-body refinement is also an important tool when fitting models into electron microscopy envelopes (see, for example, Navaza *et al.*, 2002 ▶, and numerous references therein). Rigid-body refinement may also be a way of performing coordinate refinement when only very low resolution data are available.

Rigid-body refinement moves groups of atoms as a whole, leaving the internal configuration of each group unchanged. It is well understood that the information about the large-scale distribution of atoms is contained in the low-resolution diffraction data. The high-resolution data convey information about the finer details of the atomic structure. Since these details are invariant during rigid-body refinement, it is expected that high-resolution data will be less important for this procedure than the low-resolution data. Inclusion of high-resolution data is known to hamper the progress of refinement (see, for example, Sheldrick, 2008 ▶; Sheldrick & Schneider, 1997 ▶; Tronrud, 2004 ▶). Least-squares refinement (LS) is expected to be more affected than maximum-likelihood refinement (ML), since the latter is designed to automatically weight down terms with poor model-to-data correspondence (Lunin *et al.*, 2002 ▶), *i.e.* data at high resolution at the beginning of refinement.

When choosing the high-resolution cutoff for refinement, a practical balance between convergence radius, accuracy of the results and computational cost has to be found. Generally, moving the cutoff to lower resolution is expected to increase the radius of convergence, but at the cost of decreased accuracy. This suggests a multiple resolution approach with several sequential refinements using data at increasingly higher resolution, for example, as implemented by the STIR option in *SHELX* (Sheldrick & Schneider, 1997 ▶). At the initial stage the convergence radius is large. The model is most likely to be moved closer to the correct position and orientation, but the accuracy is relatively low. At the subsequent stages the convergence radius is less critical, but the accuracy is improved by the inclusion of higher-resolution data. This approach can be robust but computationally expensive and requires *ad hoc* decisions about high-resolution data cutoff and the number of higher-resolution reflections to be added as the refinement progresses. Here we report the results of numerical experiments aimed at finding a computationally economical and automated multiple-zone refinement protocol that still results in a large convergence radius.

The multiple-zone refinement protocol is implemented in *phenix.refine* – a macromolecular structure refinement program (Afonine *et al.*, 2005*b* ▶) that is under active development as part of the *PHENIX* project (Adams *et al.*, 2002 ▶). Major development goals are increased automation and fast exploration of new approaches based on a modular architecture. Available features, among others, include various refinement targets (maximum likelihood, twinned least squares, phased maximum-likelihood), refinement of individual coordinates and ADPs (isotropic, anisotropic, group, TLS or any combination), automatic water picking built in to the refinement, robust bulk-solvent correction (Afonine *et al.*, 2005*a* ▶), Cartesian dynamics, simulated annealing, NCS restraints, refinement at ultra-high resolution (Afonine *et al.*, 2004 ▶, 2007 ▶), and joint refinement using X-ray and neutron data. Here we describe the systematic investigation of rigid-body refinement based on a large number of trial refinements in *phenix.refine*.

## 2. Methods

### 2.1. Parameterization of rigid-body motions

A rigid body is a group of atoms subject to a concerted motion, leaving the atoms fixed relative to each other. In rigid-body refinement, a macromolecule is split into one or more non-overlapping rigid groups. The position of each rigid body is characterized by six degrees of freedom. The body translation is universally parameterized as three Cartesian or fractional coordinates. The body orientation is usually defined by three Euler angles. A large number of Euler angle conventions are in use (Urzhumtseva & Urzhumtsev, 1997 ▶; Weisstein, 2006 ▶). The Euler angles are commonly referred to as α, β, γ. One commonly used convention [*e.g. AMoRe* (Navaza, 2001 ▶) and *REFMAC* (Collaborative Computational Project 4, Number 4, 1994 ▶; Murshudov *et al.*, 1997 ▶)] is to first rotate around the Cartesian *z* axis by the angle γ, then around the *y* axis by the angle β, and finally around the *z* axis again by the angle α; in this paper we refer to this convention as the *zyz* convention. At the usual starting point for rigid-body refinement, α = β = γ = 0°, α and γ are perfectly correlated, which could potentially lead to numerical instabilities. Another convention in common use (*e.g.* Urzhumtsev *et al.*, 1989 ▶; Brünger *et al.*, 1998 ▶; Kronenburg, 2004 ▶) differs in this respect. The first two rotations are as before, but the third rotation is around the *x* axis. Here we refer to this convention as the *xyz* convention. α and γ are perfectly correlated only if γ = ±90°, values that are highly unlikely to be reached in the course of rigid-body refinement as the final rotations from the starting position are typically less than 20°.

### 2.2. Refinement procedure

In the tests reported below, a rigid-body refinement run is a series of macro cycles in each of which a bulk-solvent correction is followed by L-BFGS minimization (Liu & Nocedal, 1989 ▶) (the same minimizer is used in the *CNS* program) with a maximum of 25 iterations per macro cycle. During minimization, an LS or an ML target function [implemented as defined by Lunin & Skovoroda (1995 ▶) and Afonine *et al.* (2005*a* ▶)] is used. All geometry restraints including nonbonded interactions are disabled. Thus the refinement is purely based on the experimental data.

It has been shown that a bulk-solvent correction of the low-resolution data is very important to achieve optimal refinement results (Jiang & Brünger, 1994 ▶; Kostrewa, 1997 ▶; Badger, 1997 ▶). In the tests reported below, we used the bulk-solvent correction algorithm as described by Afonine *et al.* (2005*a* ▶). In the context of rigid-body refinement, the model shifts are expected to be large and hence invalidate the bulk solvent mask calculated from the initial model. Therefore the bulk-solvent correction is tightly integrated into the refinement and recomputed between macro-cycles if the model has moved beyond a certain default threshold.

### 2.3. Test data and models

Test data and models were taken from an in-house library of 56 structures collected over the course of some time. Table 1 ▶ lists reference information for all test structures. The original experimental data were used in all trial refinements reported below. To better approach typical practical situations, the models from the library were modified by deleting all atoms that are not part of a protein, RNA or DNA molecule.

As a last manipulation, all structures were subject to rigid-body refinement using data up to 3 Å (or the high-resolution limit shown in Table 1 ▶) with the entire model as one body. The refined corrections were typically very small. These refined models were considered as the best possible results and used as the ideal (reference) model for subsequent comparisons.

For tests with multiple rigid-bodies, seven out of the 56 structures were split into two to six bodies as indicated in Table 1 ▶ (column NB). We included calmodulin and gene-5 models even though they both consist of only one chain. Calmodulin was chosen because of the space group (*P*1), gene-5 because of the small size. In each model, the chain was split into two parts and a few atoms were deleted to avoid clashes of the two artificially created bodies.

One of the multi-body structures (1071B) was subject to multi-body rigid-body refinement at 3 Å to obtain a specific multi-body reference model, since the displacements with respect to the one-body model were significant.

### 2.4. Random displacements

Our goal was to systematically sample the behavior of rigid-body refinements. For this we investigated three main variables:

(i) Averaging out model shape-related effects by using a large number of models (see previous section).

(ii) Sampling a matrix of displacement magnitudes, using combinations of translations with a 0, 2, 4 and 6 Å shift along a random vector and a 0, 5, 10 and 15° rotation around a random axis.

(iii) Averaging out effects due to interactions of model shape and translation vectors or rotation axes by sampling a large number (we used 100) of random vectors and axes for a given pair of displacement magnitudes.

The combination (translation, rotation) = (0 Å, 0°), *i.e.* no change in the starting model position or orientation, was excluded. To reduce the runtime for the tests, we also chose to omit the (4 Å, 10°), (6 Å, 10°) and (6 Å, 15°) combinations since the success rate (see §2.5 for the definition) for such very large displacements was known to be near zero, on the basis of preliminary trials. This left 12 combinations to be sampled 100 times for each of the 56 test structures, *i.e.* a total of 67 200 rigid-body refinement runs per set of trial parameters. When determining the random translations, continuous allowed origin shifts (Grosse-Kunstleve, 1999 ▶) (*e.g.* parallel to the twofold axis in space group *P*2) were specifically taken into account: the translation vectors for the first body were chosen perpendicular to the allowed origin shifts. In space group *P*1 translations of the first body have no effect on the structure factor magnitudes and were therefore not considered.

In the tests with multiple rigid bodies, the random translations and rotations can lead to serious clashes, which are unlikely to occur in most practical situations since most molecular replacement programs generally suppress configurations with clashes. However, since nonbonded interactions are not included in our rigid-body refinement procedure (§2.2), the clashes have no direct effect and we decided to ignore them.

### 2.5. Success rates

**Figure 1:**
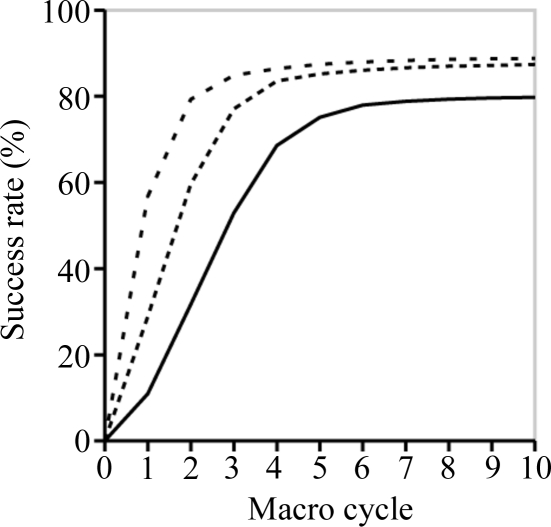
Example of a success rate plot. The horizontal axis designates the number of refinement macro cycles and the vertical axis designates the success rate in percent (see §2.5). The solid line is the plot using a 0.25 Å r.m.s.d. threshold as the criterion for ‘success’, the dashed line with shorter segments is the plot using a 0.5 Å threshold, and the dashed line with the longer segments is the plot using a 1.0 Å threshold. The example plot was obtained for rnase-p with a fixed 6.0 Å high-resolution cutoff for the data, a random translational displacement magnitude of 2.0 Å and a random rotational displacement magnitude of 5°.

After each rigid-body refinement run, the root-mean-square deviation (r.m.s.d.) with respect to the reference pre-refined model (§2.3) was determined, taking allowed origin shifts into account. For each set of 100 random displacement magnitudes (previous section), we counted the number of refined models with an r.m.s.d. less than or equal to 1.0, 0.5 and 0.25 Å. These numbers are the success rates in percent, given the chosen r.m.s.d. value. Fig. 1 ▶ shows an example plot of the success rates.

When evaluating the effects of parameter changes, we compared the success rates using a tolerance to eliminate noise. Success rates with differences less than or equal to 2% were considered insignificant. Larger differences were considered significant and used as a guide in the optimization of the refinement protocol.

## 3. Results

### 3.1. Refinements with fixed high-resolution cutoffs

Our initial test series was a systematic sampling of high-resolution cutoffs *d*_min_ = 3, 4, 6, 8 and 10 Å. Some data sets had an insufficient number of reflections given the 8 or 10 Å cutoffs. The largest resolution cutoffs used in these tests are 6 Å for gene-5 and hipip, 8 Å for gpatase, lysozyme, oat-gabaculine and rnase-p, and 10 Å for all other structures. The total number of rigid-body refinement runs over all five resolution ranges was 32 6400 = (50 structures × 5 resolution cutoffs + 2 structures × 3 resolution cutoffs + 4 structures × 4 resolution cutoffs) × 12 rotation–translation shifts × 100 trials. The complete test series was run twice: once using the LS target function, then again using the ML target function. We manually reviewed the resulting 2 × 3264 success rate plots, where each plot was an average over 100 trials.

**Figure 2:**
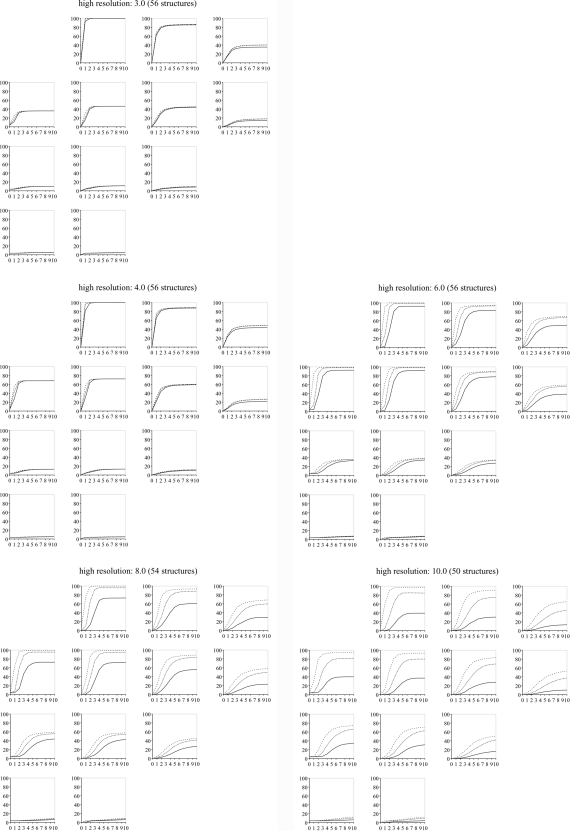
Success rate plots using the LS target function. The high-resolution values are in ångströms. The four-by-four grid for each high-resolution cutoff is arranged by rotational displacement magnitude in the horizontal direction from left to right (0, 5, 10, 15°), and translational displacement magnitude in the vertical direction downwards (0, 2, 4, 6 Å).

While there are significant individual differences between the test structures, the results show a general trend. This observation led us to prepare plots averaging the success rates over all structures (Figs. 2 ▶ and 3 ▶) so that each point in these plots shows the success rate of 100 × 56 refinements (with a few refinements less at 8 and 10 Å as explained above). This leads to the following observations:

(i) The success rates reach a plateau after a certain number of macro cycles. The height of the plateau depends on both the displacement magnitude and the high-resolution cutoff. The larger the displacement magnitudes, the lower the plateau. The larger the high-resolution cutoff, the higher the plateau.

(ii) The macro cycle at which the plateau is reached depends on the high-resolution cutoff. The larger the high-resolution cutoff, the more macro cycles are needed to reach the plateau.

(iii) The difference in the plateau heights for the three success rate cutoffs (1.0, 0.5, 0.25 Å) strongly depends on the high-resolution cutoff. With a high-resolution cutoff of 3.0 Å, the three plateaus in each plot have virtually identical heights. This suggests that, if a refinement converges to the solution, it is highly likely to be accurate. As the high-resolution cutoff is increased, the plateaus are at increasingly different heights. This means on average the solutions are increasingly less accurate.

(iv) Increasing the high-resolution cutoff leads to a larger convergence radius. This effect is most pronounced for translational displacements when going from a 3.0 to a 4.0 Å high-resolution cutoff, or from 4.0 to 6.0 Å. Larger high-resolution cutoffs do not significantly increase the convergence radius. Furthermore, the effect is weaker for rotational displacements.

(v) The difference in results obtained with the least-squares target and the maximum-likelihood target are subtle. Comparing Figs. 2 ▶ and 3 ▶ we observed that the least-squares target leads to slightly better success rates using high-resolution cutoffs of 6.0 Å or larger, and the maximum-likelihood target is slightly better using high-resolution cutoffs of 3.0 and 4.0 Å.

### 3.2. Multiple-zone protocol

The observations reported in the previous section lead to the use of a multiple-zone protocol. The goal is to take advantage of the larger convergence radius at larger high-resolution cutoffs and higher accuracy at smaller resolution cutoffs. The multiple-zone protocol automates refinement starting with a small number, n_ref(1), of low-resolution reflections (first zone), and successive addition of reflections up to a user-defined high-resolution cutoff *d*_min_. A straightforward approach is to decrement the high-resolution cutoff *d*_min_ by a certain amount after each round of rigid-body refinement, similar to the *SHELX* STIR option. Under this scheme the zones increase in size with the cube of the number of reflections. We chose to use a similar but more tunable function with a parameterization that is designed to be independent of the structure to be refined: 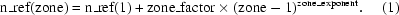
zone_exponent is a user-defined value. The zone_factor is computed from a user-defined number of zones, n_zones, and the number of reflections at the highest resolution cutoff, n_ref(n_zones): 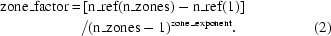
zone_exponent = 3 corresponds to the *SHELX* STIR option. With a smaller value the function is more linear, adding more reflections more quickly. With a larger value fewer reflections are added initially and more reflections in the later steps.

n_ref(1) is determined using the formula 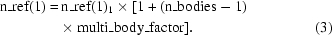
Here n_ref(1)_1_ is a user-supplied value, n_bodies is the number of user-supplied atom selections for the rigid bodies and multi_body_factor is a tunable parameter. With multi_body_factor = 1 the number of reflections for the first resolution zone is a linear function of the number of rigid bodies. The *phenix.refine* program provides two alternatives for determining n_ref(1)_1_. The user can simply specify the value directly or specify a low-resolution cutoff from which n_ref(1)_1_ is computed. In all cases, default values are automatically chosen by the program, which can be overridden by user-defined values if required.

#### 3.2.1. Exploration of parameter space

The parameterization presented in the previous section is designed to be independent of the structure to be refined. The critical variables are the number of zones n_zones, n_ref(1)_1_, the multi_body_factor and the zone_exponent. These values were optimized with a series of tests, using starting values derived from the results of the tests described above. To decrease the runtime requirements for a test series, we reviewed the refinements with fixed high-resolution cutoffs. In addition to the seven multi-body refinements, three single-body refinements were chosen with the aim of covering the distribution of number of atoms *versus* high-resolution limit of the diffraction data. The three selected structures are marked in Table 1 ▶, column NB.

A preliminary set of test runs with only seven structures split into multiple bodies indicated that n_zones = 5, zone_exponent = 4, multi_body_factor = 1 and n_ref(1)_1_ = 100 is a good default parameterization. After adding the three single-body refinements, we then started a second set of tests exploring the parameter space around these values. The results are reported in some detail below. Unless noted otherwise, the maximum-likelihood target was used in all resolution zones.

To determine the best choice for n_ref(1)_1_ we ran a test series with trial values 60, 80, 100, 120, 200 and 400. The values were chosen on the basis of the behavior of the trial refinements. Table 2 ▶ shows a mutual comparison of the n_ref(1)_1_ values of each trial with the others. Inspection suggests that the value 100 is the best choice overall for n_ref(1)_1_.

After identifying n_ref(1)_1_ = 100 as the best value, we explored values for multi_body_factor = 0.5, 1 and 2, fixing n_zones = 5 and zone_exponent = 4 as before. The results in Table 3 ▶ confirm our expectation that a simple linear coupling (multi_body_factor = 1) of the number of observations and the number of refineable parameters is optimal.

Fixing n_ref(1)_1_ = 100, multi_body_factor = 1 and n_zones = 5, we tried the alternative values zone_exponent = 1, 2, 3, 5 and 6. The results in Table 4 ▶ show that zone_exponent = 3 is the best choice. This result validates the approach used in the *SHELX* STIR algorithm.

Fixing n_ref(1)_1_ = 100, multi_body_factor = 1 and zone_exponent = 3, we tried the alternative values n_zones = 3, 4, 6, 7, 8 and 9. The disadvantage of using more zones is increased runtime. However, the results are expected to be better if more zones are used. This is largely confirmed by the data in Table 5 ▶. The use of more than five zones does not greatly improve the outcome compared with the use of three to five zones. Table 6 ▶ shows runtime statistics as a function of the number of zones. For example, using seven zones instead of five zones increases the runtime, on average, by about 25%. Thus, using five zones is a practical compromise between runtime considerations and expected benefit. However, for difficult cases it could be worth increasing the number of zones in order to increase the expected success rate, at the cost of increased runtime. It should also be noted that as rigid-body refinement is often typically only performed once at the start of structure refinement investing a some additional computing time to obtain the best solution might be well justified.

### 3.3. Effect of rotation convention

To analyze the role of different ways to describe the rotation we made a comparative refinement in similar conditions using the two different Euler angle conventions. Table 7 ▶ shows the success rate comparison using the two different Euler angle conventions introduced in §[Sec sec2.1]2.1. All other parameters were fixed at the defaults [n_ref(1)_1_ = 100, multi_body_factor = 1, zone_exponent = 3 and n_zones = 5]. The results are surprisingly clear: the *xyz* convention drastically outperforms the *zyz* convention. Even though the L-BFGS minimizer used in the refinements is designed to tolerate singularities, it is evidently advantageous to avoid them.

### 3.4. Automatic switching between least-squares and maximum-likelihood target functions

In §[Sec sec3.1]3.1 it was found that the best choice of target function depends on the high-resolution cutoff. To take advantage of this knowledge a target_auto_switch_resolution parameter was introduced. For zones with a high-resolution cutoff larger than the value of this parameter, the LS target is used, and the ML target otherwise. With optimal values for the other parameters as presented in the previous sections, a new series of four tests were performed, with target_auto_switch_resolution = 4, 5, 6 and 7 Å. The corresponding success rate comparisons are shown in Table 8 ▶. These data indicate that switching at a lower value for the resolution cutoff is better than switching at a higher value. On the basis of the fixed resolution cutoff results (Figs. 2 ▶ and 3 ▶), 6 Å was selected as the default parameter.

## 4. Conclusion

Refinement of an atomic model as a rigid body or several independent rigid bodies is an important and routine step in macromolecular refinement. By combining multi-zone rigid body refinement, robust bulk solvent and scaling, maximum likelihood methods, and large-scale optimizations of key parameters of the multi-zone protocol, it has been possible to increase the radius of convergence of rigid-body refinement and make the process highly automated.

We observe that although likelihood methods do provide for some degree for automated weighting of data the extent of this weighting is not sufficient to provide a large radius of convergence when high-resolution data are used in the rigid-body refinement. Thus, explicit removal of higher-resolution data during rigid-body refinement, even when using a likelihood target, significantly increases the radius of convergence.

The idea of gradually increasing the refinement resolution from low to high or simply truncating the high-resolution data at some point between 3 and 6 Å resolution has been used by many practitioners for some time. However, the systematic investigation undertaken here has provided two very important enhancements. First, use of the number of reflections to define the refinement resolution zones, instead of specific resolution limits, makes the process model-independent. For example, for relatively small structures cutting the data at 6 Å or even higher may not leave enough low-resolution reflections for refinement. In addition, defining the zones by the number of reflections always assures an adequate amount of data and an appropriate resolution for the refinement. Second, since large model shifts are expected during rigid-body refinement, it is essential to update the bulk solvent model as often as a model shifts beyond a certain threshold or additional reflections are included.

Having performed hundreds of thousands of rigid-body refinements using a set of 56 models different in size, shape and packing, as well as having different quality experimental data sets associated with them (resolution and completeness), we have empirically confirmed our hypothesis that the *xyz* rotation parameterization performs better than the *zyz* parameterization since it avoids a singularity near the typical values for the rotation parameters encountered in refinement. Our results also define approximate convergence radii for gradient-driven rigid-body refinement (Figs. 2 ▶ and 3 ▶).

Our systematic exploration of the parameter space (§3.2.1) was feasible only because we had access to a computer cluster with 200 fast CPUs. The tools that we have developed for running and analyzing the many refinements are being re-used for evaluating other algorithms and parameterizations. By making use of the latest computing technology, we can replace the very slow and subjective process of tuning parameters based on anecdotal evidence with a more scientific approach. With modern tools, practical experience that may have taken many years to accumulate in the past can now be obtained in a matter of days.

The described algorithms and protocols are implemented in the refinement program *phenix.refine*, which is available as part of the *PHENIX* package. The program is available from http://www.phenix-online.org/. The core rigid-body calculations are part of open source libraries (http://cctbx.sourceforge.net).

## Figures and Tables

**Figure 3:**
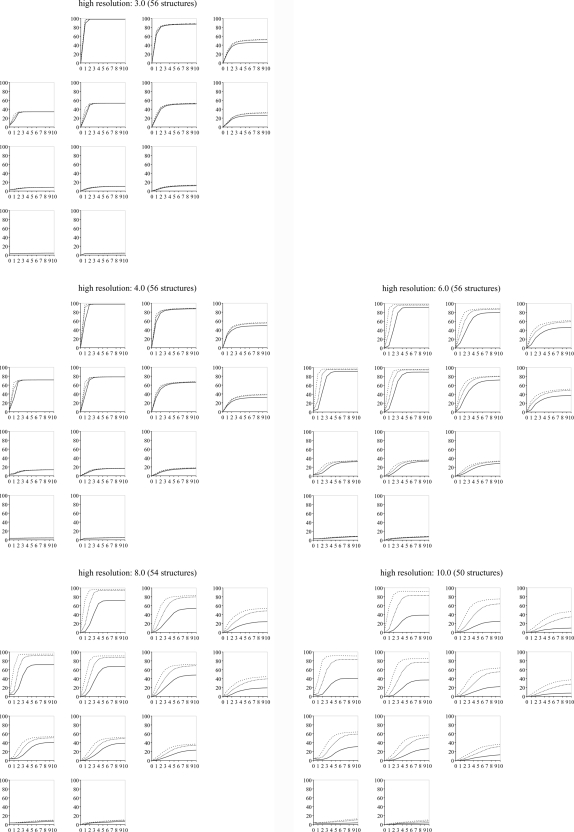
Success rate plots using the ML target function. See the caption of Fig. 2 ▶ for a guide to the plots.

**Table 1:** Overview of structures used in tests Resol. is the high-resolution limit (Å) of the observed data. NA is the number of atoms used in refinement (protein and nucleic acid only). The PDB ID column refers to related Protein Data Bank (Berman *et al.*, 2000 ▶) entries with the same space group and a similar unit cell. In some cases, the data and model deposited in the PDB are slightly different from those used in the tests. NB is the number of bodies and marks the ten structures used in the second test series (see §[Sec sec2.3]2.3).

Database ID	Resol.	NA	PDB ID	NB
group2-intron	3.5	1497	1kxk	1
synaptotagmin	3.2	2186	1dqv	
1029B	3.0	9230	1n0e	
1038B	3.0	11038	1lql	5
1071B	3.0	6558	1nf2	6
proteasome	2.9	24927	1q5q	
sec17	2.9	2217	1qqe	
cp-synthase	2.8	4331	1l1e	
penicillopepsin	2.8	2366	3app	
s-hydrolase	2.8	6666	1a7a	
ut-synthase	2.8	7504	1e8c	
gere	2.7	3060	1fse	
groel	2.7	26957	1oel	
aep-transaminase	2.6	16698	1m32	4
rab3a	2.6	2431	1zbd	
a2u-globulin	2.5	5148	2a2u	4
flavin-reductase	2.5	3385	1bkj	
p32	2.5	4265	1p32	
psd-95	2.5	2180	1jxm	
qaprtase	2.5	12570	1qpo	1
rnase-s	2.5	1488	1rge	
1102B	2.5	2662	1l2f	
rh-dehalogenase	2.45	2336	1bn7	
armadillo	2.4	3458	3bct	
cyanase	2.4	11970	1dw9	
fusion-complex	2.4	7025	1sfc	
human-otc	2.4	2528	1ep9	
mev-kinase	2.4	2506	1kkh	
nsf-d2	2.4	1943	1nsf	
granulocyte	2.35	1908	2gmf	
oat-gabaculine	2.3	9450	1gbn	2
vmp	2.3	7992	1l8w	
gpatase	2.25	7786	1ecf	
hn-rnp	2.2	1338	1ha1	
antitrypsin	2.1	2985	1hp7	
pdz	2.1	1372	1kwa	
1167B	2.0	2920	1s12	
apoferritin	2.0	1354	1gwg	
cobd	2.0	2738	1lkc	
synapsin	2.0	4636	1auv	1
tryparedoxin	2.0	1145	1qk8	
myoglobin	1.9	1227	1n9x	
nsf-n	1.9	1518	1qcs	
rop	1.9	850	1f4n	
epsin	1.8	1210	1edu	
gene-5	1.8	673	1vqb	2
ic lyase	1.8	6484	1f61	
mbp	1.8	1760	1ytt	
p9	1.75	1062	1bkb	
1063B	1.7	1926	1lfp	
nitrite-reduct	1.7	2582	1et7	
insulin	1.7	400	2bn3	
lysozyme	1.5	982	1aki	
rnase-p	1.5	3607	1nz0	
calmodulin	1.1	1150	1exr	2
hipip	0.8	616	1iua	

**Table 2:** Comparison of success rates for different values of the n_ref(1)_1_ parameter (§3.2.1) The first row and the first column show the parameter values. The diagonal and the redundant lower triangle are omitted. Each cell shows three triples of success rates, for the r.m.s.d. cutoffs 1.0 Å (first row), 0.5 Å (second row) and 0.25 Å (third row), respectively. The left value in each triple is the number of times the success rate obtained with the parameter value given by the corresponding row was at least 2% better than that with the parameter value given by the corresponding column (§2.5); the right value is the number of times the success rate obtained with the parameter value given by the corresponding column was at least 2% better than that with the parameter value given by the corresponding row; the value in the middle is the number of times the difference between the success rates was smaller than 2%.

n_ref(1)_1_	80	100	120	200	400
60	(26, 66, 28)	(25, 62, 33)	(28, 54, 38)	(30, 56, 34)	(38, 49, 33)
	(26, 65, 29)	(25, 63, 32)	(28, 53, 39)	(30, 56, 34)	(39, 48, 33)
	(26, 71, 23)	(25, 68, 27)	(27, 59, 34)	(27, 59, 34)	(34, 54, 32)
					
80		(24, 72, 24)	(27, 63, 30)	(35, 53, 32)	(39, 55, 26)
		(23, 73, 24)	(26, 64, 30)	(35, 54, 31)	(39, 55, 26)
		(21, 77, 22)	(23, 67, 30)	(29, 60, 31)	(32, 62, 26)
					
100			(27, 69, 24)	(34, 66, 20)	(42, 55, 23)
			(27, 69, 24)	(33, 67, 20)	(41, 56, 23)
			(25, 73, 22)	(27, 73, 20)	(35, 63, 22)
					
120				(28, 58, 34)	(37, 55, 28)
				(28, 58, 34)	(37, 55, 28)
				(23, 64, 33)	(31, 62, 27)
					
200					(33, 66, 21)
					(34, 65, 21)
					(31, 69, 20)

**Table 3:** Comparison of success rates for different values of the multi_body_factor parameter (§3.2.1) See caption of Table 2 ▶ for a guide to the data in this table. However, in this case the count in the middle of each triplet is given as a sum of two values: the first value is for zones that are different; the second value is for zones that are not affected by the parameter and therefore lead to exactly identical results [see equations (1)–(3) [Disp-formula fd3] in §3.2; in this case the three one-body structures are insensitive to the multi_body_factor].

multi_body_factor	1.0	2.0
0.5	(15, 44+36, 25)	(15, 36+36, 30)
	(15, 45+36, 24)	(15, 36+36, 30)
	(15, 50+36, 19)	(14, 42+36, 25)
		
1.0		(17, 50+36, 14)
		(18, 49+36, 14)
		(15, 52+36, 14)

**Table 4:** Comparison of success rates for different values of the zone_exponent parameter (§3.2.1) See caption of Table 2 ▶ for a guide to the data in this table.

zone_exponent	2	3	4	5	6
1	(5, 73, 42)	(6, 60, 54)	(13, 54, 53)	(19, 59, 42)	(20, 62, 38)
	(5, 73, 42)	(6, 60, 54)	(13, 55, 52)	(19, 59, 42)	(21, 61, 38)
	(5, 77, 38)	(6, 69, 45)	(13, 62, 45)	(19, 67, 34)	(21, 68, 31)
					
2		(14, 63, 43)	(15, 70, 35)	(20, 74, 26)	(27, 66, 27)
		(14, 63, 43)	(15, 71, 34)	(20, 74, 26)	(27, 66, 27)
		(13, 71, 36)	(15, 76, 29)	(22, 77, 21)	(25, 73, 22)
					
3			(33, 62, 25)	(34, 71, 15)	(34, 75, 11)
			(33, 62, 25)	(34, 72, 14)	(34, 76, 10)
			(28, 67, 25)	(31, 75, 14)	(32, 78, 10)
					
4				(30, 77, 13)	(34, 72, 14)
				(31, 76, 13)	(34, 71, 15)
				(30, 79, 11)	(32, 74, 14)
					
5					(23, 74, 23)
					(24, 73, 23)
					(20, 79, 21)

**Table 5:** Comparison of success rates for different values of the n_zones parameter (§3.2.1) See caption of Table 2 ▶ for a guide to the data in this table.

n_zones	4	5	6	7	8	9
3	(14, 68, 38)	(2, 67, 51)	(10, 60, 50)	(9, 53, 58)	(8, 50, 62)	(7, 52, 61)
	(13, 68, 39)	(2, 66, 52)	(10, 60, 50)	(9, 53, 58)	(8, 50, 62)	(7, 51, 62)
	(13, 73, 34)	(2, 74, 44)	(9, 65, 46)	(9, 61, 50)	(8, 57, 55)	(7, 59, 54)
						
4		(7, 70, 43)	(9, 70, 41)	(7, 65, 48)	(4, 59, 57)	(4, 62, 54)
		(7, 70, 43)	(10, 68, 42)	(7, 65, 48)	(4, 59, 57)	(4, 62, 54)
		(6, 75, 39)	(8, 73, 39)	(7, 69, 44)	(3, 65, 52)	(4, 68, 48)
						
5			(23, 73, 24)	(17, 76, 27)	(15, 70, 35)	(14, 68, 38)
			(24, 72, 24)	(17, 76, 27)	(15, 70, 35)	(14, 69, 37)
			(19, 77, 24)	(16, 77, 27)	(13, 74, 33)	(15, 73, 32)
						
6				(18, 70, 32)	(11, 71, 38)	(13, 71, 36)
				(18, 70, 32)	(11, 71, 38)	(13, 71, 36)
				(17, 76, 27)	(11, 74, 35)	(13, 77, 30)
						
7					(18, 73, 29)	(17, 79, 24)
					(17, 74, 29)	(16, 80, 24)
					(14, 79, 27)	(16, 84, 20)
						
8						(19, 87, 14)
						(20, 85, 15)
						(20, 90, 10)

**Table 6:** Comparison of runtimes for different values of the n_zones parameter (§3.2.1) The runtime statistics shown in each row (columns 2–4) are based on 10 × 12 values (number of test structures × number of displacement combinations). Columns 5–10 show the ratios of the mean runtimes (mean in the given row divided by mean in the previous rows).

	Runtime (s)			n_zones					
n_zones	Minimum	Maximum	Mean	3	4	5	6	7	8
3	15.06	571.2	222.567						
4	16.69	717.6	263.241	1.18					
5	17.77	795.6	295.355	1.33	1.12				
6	19.50	879.6	330.116	1.48	1.25	1.12			
7	21.61	1018.2	370.33	1.66	1.41	1.25	1.12		
8	21.82	1063.8	407.154	1.83	1.55	1.38	1.23	1.10	
9	23.28	1191.6	438.968	1.97	1.67	1.49	1.33	1.19	1.08

**Table 7:** Comparison of success rates using the two Euler angle conventions (§§2.1 and 3.3) See caption of Table 2 ▶ for a guide to the data in this table.

Convention	*zyz*
*xyz*	(75, 40, 5)
	(91, 25, 4)
	(83, 35, 2)

**Table 8:** Comparison of success rates using different values for the resolution at which the target function is switched from least squares to maximum likelihood (§3.4) See caption of Table 3 ▶ for a guide to the data in this table.

Switch resolution (Å)	5	6	7
4	(1, 56+60, 3)	(7, 102, 11)	(7, 102, 11)
	(0, 57+60, 3)	(5, 102, 13)	(5, 102, 13)
	(0, 57+60, 3)	(5, 102, 13)	(5, 102, 13)
			
5		(8, 92+12, 8)	(8, 92+12, 8)
		(7, 91+12, 10)	(7, 91+12, 10)
		(6, 92+12, 10)	(6, 92+12, 10)
			
6			(0, 12+108, 0)
			(0, 12+108, 0)
			(0, 12+108, 0)
